# Evaluating the Relationship Between Online Learning Environment and Medical Students’ Wellbeing During COVID-19 Pandemic

**DOI:** 10.21315/mjms2021.28.5.11

**Published:** 2021-10-26

**Authors:** Rita Mustika, Edward Christopher Yo, Muhammad Faruqi, Rahma Tsania Zhuhra

**Affiliations:** 1 Medical Education Center, Indonesian Medical Education and Research Institute (IMERI), Universitas Indonesia, Jakarta, Indonesia; 2 Medical Education Collaboration Cluster, Indonesian Medical Education and Research Institute (IMERI), Universitas Indonesia, Jakarta, Indonesia; 3 Department of Medical Education, Faculty of Medicine, Universitas Indonesia, Jakarta, Indonesia

**Keywords:** COVID-19, medical students, online learning environment, wellbeing

## Abstract

**Background:**

The coronavirus disease 2019 (COVID-19) pandemic has been found to negatively affect medical students’ wellbeing. This finding may be related to how medical education is being conducted at present, with online learning replacing face-to-face teaching in many countries. This cross-sectional study aims to assess how the online learning environment is connected to medical students’ wellbeing.

**Methods:**

A self-administered online questionnaire was distributed to undergraduate medical students at Universitas Indonesia. The study was conducted from September 2020 to February 2021. The questionnaire included a modified version of the Online Learning Environment Scale (OLES) and the Positive Emotion, Engagement, Relationships, Meaning and Accomplishment (PERMA) profiler. The OLES was used to evaluate students’ perceptions of the online learning environment, whereas the PERMA Profiler was used to evaluate students’ wellbeing. We validated the questionnaire before distribution. The content validity index was 1.0, with internal consistency coefficients of 0.87 and 0.89, respectively. Regression analyses were performed to evaluate the relationship between OLES and PERMA scores.

**Results:**

The questionnaire was completed by 274 undergraduate medical students. Students reported moderate to high degrees of positive perception towards online learning, high levels of positive emotions and moderate levels of negative emotions. Statistically significant differences were found across groups based on students’ gender, year of study and academic programme. Almost all aspects of the online learning environment were significantly predictive of students’ wellbeing, with personal relevance and evaluation and assessment being the two most important predictors (*R*^2^ = 0.201; *P* < 0.001).

**Conclusion:**

Medical students generally enjoyed online learning, although some challenges were presented. The online learning environment was positively associated with students’ wellbeing; however, some students expressed negative emotions including loneliness, anxiety, anger and sadness.

## Introduction

Despite countless pandemic control measures during the past year, coronavirus disease 2019 (COVID-19) remains a global threat that may undermine an individual’s quality of life. The disease, which is caused by the highly pathogenic respiratory virus, severe acute respiratory syndrome coronavirus 2 (SARS-CoV-2), has claimed more than 2,527,000 lives worldwide (1). The pandemic has caused unprecedented psychological and social devastation for many individuals, particularly healthcare workers. Previous reports confirmed that healthcare workers, including physicians, who treated COVID-19 patients suffered from high levels of stress, anxiety, sleep deprivation and depression — all of which have been negatively associated with the state of wellbeing (2–3).

Wellbeing is often defined as the state in which an individual possesses the adequate physical, psychological and social resources to meet a particular challenge (4). Not only physicians but also medical students have been affected by lower wellbeing during the pandemic. The amount of time spent during online learning may be similar to that spent during face-to-face learning on a typical day, but an increasing number of students claim to be more stressed than usual. This condition, termed ‘videoconferencing fatigue’ by some, is considered by several studies to interfere with learners’ wellbeing (5–6). Lyons et al. (7) found that the uncertainty arising from abrupt changes to medical courses (e.g. online learning, elimination of face-to-face discussions) has contributed to higher psychological distress among medical students. These changes in communication may create a sense of disconnect between teachers and students as well as between students. Hence, as online learning continues, students may be exposed to greater risk of anxiety, loneliness and feelings of isolation (5, 8).

According to wellbeing theory (9), several elements contribute to students’ wellbeing, including positive emotions, engagement, relationships, meaning and accomplishments. A study in Malaysia found that connectedness or close relationships that depend on face-to-face interaction and direct interpersonal relationships had the greatest influence on wellbeing (10). However, since face-to-face interaction is not feasible due to pandemic constraints, medical schools must consider alternative ways of creating a comfortable and inclusive online learning environment.

Poor wellbeing in medical students is a significant predictor of burnout and decreased resilience (11). In other words, students with good wellbeing are more likely to become compassionate, resilient and empathetic physicians in the future. If not acted upon promptly, personal distress may worsen throughout students’ course of medical study and continue when they become physicians (6). At present, despite the increasing burden of the pandemic on medical education, the relationship between the online learning environment and medical students’ wellbeing remains understudied. As most medical schools in the world are still heavily dependent on online learning, there is an urgent need to explore this field of research.

## Methods

### Design, Population and Sampling

This analytic, cross-sectional study was conducted in the Faculty of Medicine, Universitas Indonesia, which transformed its curriculum into fully online learning courses due to the COVID-19 pandemic. We developed the proposal in September 2020, distributed the online questionnaire in November 2020 and completed the research process in February 2021. This study’s population included second-, third- and fourth-year undergraduate medical students. We did not include first-year students in this study since they started their academic course entirely online and thus could not compare online and offline learning methods. As the Faculty of Medicine at the Universitas Indonesia has two different medical study programmes (a regular and an international programme), we included students from both programmes in the study population. The most significant difference between the two programmes is that students from the international programme study abroad for one year after completing three years of preclinical courses. In addition, there are fewer international programme students than regular programme students in a cohort; the ratio of regular programme students to international programme students is roughly 3:1. We calculated the sample size using an online sample size calculator, Statistics Kingdom, based on 90% power, a medium effect size and a 5% significance level for 12 predictors. The calculated sample size was 155. After adding 10% dropout rate, the total size needed became 170. We used total sampling in this study. Our total number of respondents was 274, which was more than the minimum sample needed.

### Instruments

We used a modified version of the Online Learning Environment Scale (OLES) to assess the online learning environment (12) as well as the Positive Emotion, Engagement, Relationships, Meaning and Accomplishment (PERMA) profiler to measure medical students’ wellbeing (13).

### Modified Online Learning Environment Scale

The original OLES questionnaire was developed by Trinidad et al. (12) with nine scales derived from other instruments to measure the online learning environment, including Distance Education Learning Environment Survey (DELES), Constructivist Learning Environment Survey (CLES), Technology-Rich Outcome-Focus Learning Environment Inventory (TROFLEI) and What is Happening in this Class (WIHIC). The nine scales include computer usage (CU), teacher support (TS), student interaction and collaboration (SIC), personal relevance (PR), authentic learning (AL), student autonomy (SA), equity (EQ), asynchronicity (AS) and enjoyment (EN). This study utilised a modified version of the OLES that was established by Yee (13) with three additional scales: i) evaluation and assessment (EA); ii) online learning tools (OLT) and iii) design interface (DI). Moreover, since medical students in our setting use computers and other electronic devices for online learning, we renamed the ‘computer usage’ scale as ‘electronic device usage’ (EDU) for this study only. The questionnaire’s statements were in the form of a five-point Likert scale ranging from 1 (never) to 5 (always). Before data collection, we validated the Indonesian version of the modified OLES on Indonesian medical students. The validation process included forward-backward translation, expert review involving five medical education experts, cognitive interviews with 20 medical students and pilot testing with 50 respondents. Congruent with the English version, the final Indonesian version of the modified OLES consisted of 12 domains using a five-point Likert scale. The content validity index (CVI) from expert review was 1.0 and the internal consistency coefficient was 0.87. Therefore, we considered the OLES valid and reliable.

### Positive Emotion, Engagement, Relationships, Meaning and Accomplishment Profiler

This instrument was initially developed by Butler and Kern (14) with five scales, including positive emotion (P), engagement (E), relationships (R), meaning (M) and accomplishment (A). Following recommendations from the authors, this study also included questions about physical health (H), negative emotion (N), loneliness (LON) and overall wellbeing (OWB) to provide more information. The OWB score was obtained by calculating the mean of the P, E, R, M, A and overall happiness scores. The statements in the questionnaire were in the form of a 10-point Likert scale ranging from 1 (never/terrible/not at all) to 10 (always/excellent/completely) (14). We used the validated Indonesian version of the PERMA profiler (15). The Indonesian version of PERMA profiler uses the same scales as the original version. To ensure the validity of the questionnaire, we performed an expert review and measured the internal consistency. The CVI was 1.0 and the internal consistency coefficient was 0.89.

### Procedure

This study was approved by the Research Ethics Committee of the Faculty of Medicine, Universitas Indonesia. Background information regarding the study was presented to the study population, and only those who gave consent and voluntarily filled out the questionnaire were included in the data analysis.

### Data Analysis

All quantitative data were analysed using the Statistical Package for Social Sciences (SPSS) software version 24. We assessed data for normality using a histogram and Kolmogorov–Smirnov test. Differences between groups were evaluated using either the independent samples *t*-test or one-way analysis of variance (ANOVA), followed by post-hoc analysis using Tukey’s Honestly Significant Different (HSD) test. We then analysed the correlation between the online learning environment and students’ wellbeing to determine the relationship between variables and to assess the strength of the relationship. Additionally, we applied multiple linear regression analysis using the enter method to evaluate the relationship between the online learning environment as assessed by OLES and students’ wellbeing.

## Results

### Demographics

A total of 274 undergraduate medical students completed the questionnaire. Participants were 62% female. The majority (92%) resided in Java Island. The proportion of second-, third- and fourth-year respondents was similar (29.2%–39.8%), although slightly more respondents were second-year students. Almost 80% were from the regular programme. Demographic information is presented in Table 1.

### Students’ Perception of the Online Learning Environment

Table 2 shows the overall mean and standard deviation for each OLES scale, which is used to describe several aspects of the online learning environment. The mean scores ranged from 3.10 (EA) to 4.87 (EDU), suggesting that students generally had moderate to high degrees of positive perception towards online learning. The low standard deviations indicate that there were no significant outliers. We found statistically significant differences between male and female students’ scores on six OLES scales (EDU, LS, AS, EA, OLT and ID). Female students scored significantly higher than male students on five of those six scales. The mean scores for the OLES scales were comparable between regular and international programme students, except for PR and AS, where students from the international programme reported statistically significant higher scores (*P* = 0.002; *P* = 0.024).

A one-way ANOVA showed statistically significant differences in six OLES scales (PR, AL, SA, EN, AS and EA) between students from Years 4, 3 and 2 (Table 3). To further investigate where the differences occurred between the three groups, a post-hoc analysis using Tukey’s HSD test was performed. Fourth-year students had significantly lower PR and AL scores than both third- and second-year students as well as significantly lower SA, EN and AS scores than second-year students. The only OLES scale where students from Year 4 scored significantly higher was EA.

### Students’ Wellbeing During Online Learning

Table 4 shows the overall mean and standard deviation for each PERMA scale, which describes students’ wellbeing within a specified time period. On the positive wellbeing scales (P, E, R, M, A, OWB and H), H received the lowest score while E was scored the highest. On the negative wellbeing scales (N, LON), the mean scores were similar. Therefore, it can be inferred from these scores that students had relatively high levels of positive emotions and moderate levels of negative emotions throughout the online learning period.

No statistically significant differences were found in PERMA scores between male and female students except for LON, where male students reported higher scores (*P* = 0.036). Students from the international programme were also found to be significantly lonelier than their peers from the regular programme (*P* = 0.032).

Differences in wellbeing scores between students from Years 4, 3 and 2 were analysed using one-way ANOVA and Tukey’s HSD test (Table 5). Third-year students had significantly higher scores for P and OWB than second-year students. Additionally, third-year students had significantly higher scores for A than fourth-year students.

### Relationship Between the Online Learning Environment and Students’ Wellbeing

We used correlation analyses and multiple linear regression to estimate relationships between the online learning environment and medical students’ overall wellbeing (Table 6). A weak to moderate correlation was found between OLES scores and students’ wellbeing. The scale least associated with wellbeing was EDU (0.044), whereas the scale most associated with wellbeing was PR (0.365).

The multiple correlation coefficient (*R*) for the 12 independent variables was 0.448. The multiple linear regression model was found to be highly significant (*P* < 0.001) and explained 20.1% of the variance (adjusted *R*^2^ = 16.4%) in students’ wellbeing. Standardised regression weights (beta) for the OLES scales were compared to determine which were the most important predictors of students’ wellbeing. Standardised regression weights rather than unstandardised weights were used, as the aim of this study was to predict significant relationships between the online learning environment and wellbeing as well as to investigate the effect of the independent variables on the dependent variable. Two scales, PR and EA, were statistically significantly (*P* = 0.019; *P* = 0.04) relative to the dependent variable (Table 6). Tolerance and the variance inflation factor (VIF) value of all variables were assessed to exclude any threat of multicollinearity that might distort the result. All tolerance values were above 0.100 (range = 0.487–0.925) and VIF values were below 5 (range = 1.081–2.053); thus, no multicollinearity was found.

## Discussion

Based on the literature, we presumed that students would be exhausted from online learning and therefore perceive it negatively. For instance, a recent study by Dost et al. (16) showed that medical students did not generally enjoy online learning and highly preferred face to-face teaching. On the contrary, our findings showed that medical students in our sample tended to enjoy online learning despite various challenges during its implementation.

Our study found that medical students at Universitas Indonesia had moderate to high mean scores on the OLES, suggesting a positive experience with various aspects of the online learning environment. In addition, results from the PERMA Profile showed that students’ wellbeing during the online learning period was relatively good. This trend is consistent with results from a study at Astana Medical University, Kazakhstan, which found that the transition from traditional learning to online learning decreased the prevalence of burnout syndrome, anxiety, depression and somatic symptoms among medical students (17).

Results from the regression model indicated that nearly all aspects of the online learning environment were important predictors of medical students’ wellbeing. Even the use of online learning tools and electronic devices can affect students’ perceptions towards online learning. Shehzadi et al. (18) argued that the quality of information and communication technology at the university positively contributed to students’ satisfaction. These findings might be related to recent generation profiles. In this study, the undergraduate students were from Gen Z (born between 1996 and 2010) (19). Eckleberry-Hunt et al. (20) stated that Gen Z students tend to use technology on a daily basis, including for learning purposes. Moreover, of the 12 OLES scales, personal relevance (PR) and evaluation and assessment (EA) stood out as the two aspects most associated with students’ wellbeing. According to Priniski et al. (21), relevance is a personal, meaningful connection to an individual and can be conceptualised along a continuum of personal meaningfulness, ranging from personal association to identification. In terms of the OLES, Trinidad et al. (12) defined personal relevance as the extent to which it is fundamental to complete planned activities and stay on the subject matter. Recent medical students, as part of Gen Z, tend to seek meaning and personalised goals in choosing and experiencing their learning (19). It can therefore be inferred from our study that students clearly wanted their online learning experience to be relevant to their learning development, and feedback received from peer or tutor evaluation and assessment can help them stay on track.

Gender differences played a role in whether students perceived the online learning environment positively. Overall, female students rated most aspects of the online learning environment higher than their male counterparts. Female students were also found to be less lonely than male students. As females were found to experience better social presence during online learning, they could have better performance and satisfaction than males (22). Women tend to have better peer-to-peer communication that helped them overcome challenges in online learning. Morante et al. (23) summarised the findings from various studies, concluding that women tend to be personal, task oriented and collaborative with their peers in online learning. They do not hesitate to establish deeper discussion, provide commentaries, share apologies and motivate one other.

Additionally, the academic cohort to which the student belonged also contributed to differences in perception and wellbeing during online learning. Senior students from Year 4 reported lower OLES scores than their juniors from Year 3 and Year 2. This might be due to the fact that fourth-year students were currently preparing to attend clinical-phase education. At the time of the survey, these senior students were in a ‘transition period’ between preclinical and clinical courses. Meanwhile, students from Years 2 and 3 were still in the pre-clinical phase of education. The fourth-year students might have been expected to put more effort and time into adapting to the new phase, and this might expose them to higher stress levels, worsened by the limitations of online learning. According to a qualitative study by Khalil et al. (24), clinical students were more interested in real classroom interaction on campus and live clinical participations. Al-Balas et al. (25) found that overall satisfaction during online learning was only 26.8% in medical students in Jordan during their clinical years. Furthermore, final-year students were concerned about the limitations of online learning in providing clinical skills and experiences, such as in bedside teaching sessions (26).

In terms of wellbeing, students from Year 3 seemed to be most content during the online learning period. These students have had enough time to settle in and become familiar with the modules, unlike second-year students who were still in the early stages of their studies. Third-year students also still have time to explore a number of opportunities, such as competitions and volunteering, unlike fourth-year students who are about to begin the clinical phase and reported lower accomplishment scores.

Meanwhile, differences between the regular and international programmes did not seem to have a significant relationship with students’ experience and welfare. The curriculums of the two programmes differ only slightly in the first year and the year abroad, and are identical in other academic years. Hence, although some significant differences in the OLES and PERMA scores were found, it is more likely that these were due to other unmeasured factors.

Despite the positive takeaways, it may be too early to be overly optimistic. The students claimed to experience moderate levels of negative emotions throughout the online learning period. These negative emotions included loneliness, anxiety, anger and sadness. This alarming trend should become a concern for medical educators, as the current pandemic situation seems to suggest that traditional offline learning may not return soon (27). Although vaccines are being prepared for dissemination to the public, deaths due to COVID-19 continue to rise. Considering the likelihood that online learning may be extended another year, medical educators must quickly find a way to deal with the negative emotions experienced by students.

However, since this study recruited a relatively small sample size from only one medical school in Indonesia, its findings may not be representative of other students from different medical faculties and countries. There are also many variations in the quality of medical education as well as the availability of teaching resources, especially technology logistics, among medical schools in Indonesia during the pandemic. Factors such as platform incapability, connection problems and faculty members’ readiness contributed to the challenges encountered by higher-education institutions, including medical schools. Future research is needed to provide more comprehensive insight into medical students’ situations and readiness for online learning. Both quantitative and qualitative research is needed to explore and develop a suitable online learning model for use by medical schools. Within the context of Indonesia, the readiness of medical schools to implement online learning effectively must also be assessed and compared, especially between institutions based in Java Island and those outside Java, where resource differences such as internet speed may become apparent.

## Conclusion

Despite the heavy burden of the pandemic on medical education, the experience of online learning among medical students was found to be positive and important to their state of wellbeing. Gender and year of study influenced students’ perception towards the online learning environment and students’ wellbeing throughout the pandemic period. Future research is needed to elaborate on ways to help students overcome negative emotions during online learning. A more comprehensive insight into the readiness of medical students as well as medical schools in preparing for online learning is also needed.

## Figures and Tables

**Table 1:** Demographic information on the subjects (*N* = 274)

Variables	Number	Percentage
Gender		
Male	104	38.0
Female	170	62.0
Location		
Java Island	252	92.0
Outside Java Island	22	8.0
Year of study		
Year 4	85	31.0
Year 3	80	29.2
Year 2	109	39.8
Programme		
Regular	217	79.2
International	57	20.8

**Table 2:** Overall mean and standard deviation for each OLES scale

OLES scales	Mean	Standard deviation
EDU	4.87	0.26
LS	4.22	0.63
SIC	4.50	0.57
PR	3.89	0.73
AL	4.02	0.65
SA	4.30	0.65
EQ	4.48	0.55
EN	3.45	0.86
AS	4.27	0.60
EA	3.10	0.68
OLT	4.51	0.52
ID	4.30	0.63

**Table 3:** OLES mean and standard deviation for students from Years 4, 3 and 2

OLES scales	Mean (SD)			*P*-value

	Year 4 students	Year 3 students	Year 2 students	
EDU	4.83 (0.32)	4.91 (0.20)	4.87 (0.24)	0.154
LS	4.24 (0.62)	4.16 (0.74)	4.14 (0.55)	0.636
SIC	4.43 (0.52)	4.54 (0.56)	4.52 (0.60)	0.375
PR	3.67 (0.67)	3.95 (0.73)	4.01 (0.74)	0.005
AL	3.80 (0.64)	4.11 (0.63)	4.13 (0.64)	0.001
SA	4.16 (0.69)	4.35 (0.62)	4.37 (0.61)	0.046
EQ	4.43 (0.54)	4.47 (0.59)	4.51 (0.52)	0.586
EN	3.20 (0.88)	3.46 (0.81)	3.64 (0.85)	0.002
AS	4.12 (0.61)	4.25 (0.59)	4.41 (0.57)	0.004
EA	3.29 (0.60)	2.95 (0.74)	3.06 (0.65)	0.003
OLT	4.50 (0.43)	4.52 (0.60)	4.50 (0.52)	0.960
ID	4.27 (0.65)	4.40 (0.66)	4.25 (0.58)	0.215

**Table 4:** Overall mean and standard deviation for each PERMA scale

Wellbeing scales	Mean	Standard deviation
P	7.40	1.52
E	7.81	1.25
R	7.43	1.54
M	7.65	1.55
A	7.55	1.11
OWB	7.57	1.18
N	5.94	1.94
H	7.11	1.69
LON	5.95	2.55

**Table 5:** PERMA mean and standard deviation for students from Years 4, 3 and 2

Wellbeing scales	Mean (SD)			*P*-value

	Year 4 students	Year 3 students	Year 2 students	
P	7.57 (1.52)	7.65 (1.28)	7.08 (1.64)	0.017
E	7.76 (1.27)	8.00 (1.18)	7.71 (1.28)	0.259
R	7.49 (1.55)	7.70 (1.34)	7.19 (1.65)	0.072
M	7.75 (1.44)	7.90 (1.25)	7.39 (1.78)	0.066
A	7.35 (1.16)	7.85 (0.94)	7.49 (1.15)	0.010
OWB	7.59 (1.15)	7.81 (1.01)	7.37 (1.29)	0.040
N	5.64 (1.99)	5.85 (2.00)	6.25 (1.82)	0.084
H	7.07 (1.83)	7.47 (1.68)	6.87 (1.55)	0.055
LON	5.68 (2.53)	6.33 (2.59)	5.89 (2.53)	0.257

**Table 6:** Correlation and multiple linear regression analysis to predict medical student wellbeing based on dimensions of the OLES

OLES dimensions	Pearson correlation coefficient (*r*)	Standardised regression weight (*β*)	*P*-value
EDU	0.044	−0.040	0.494
LS	0.115	−0.015	0.818
SIC	0.229	0.027	0.690
PR	0.365	0.187	0.019
AL	0.278	0.036	0.617
SA	0.311	0.132	0.066
EQ	0.215	0.075	0.259
EN	0.299	0.097	0.164
AS	0.218	−0.043	0.549
EA	0.205	0.117	0.043
OLT	0.117	0.015	0.812
ID	0.180	0.098	0.120

Note: *R*^2^ = 0.201 (*P* < 0.001)
